# Assembly and Comparative Analysis of the Complete Mitochondrial Genome of *Bromus inermis*

**DOI:** 10.3390/genes16060652

**Published:** 2025-05-28

**Authors:** Sibin Feng, Zinian Wu, Chunyu Tian, Yanting Yang, Wenlong Gong, Zhiyong Li

**Affiliations:** 1 Institute of Grassland Research, Chinese Academy of Agricultural Sciences, Hohhot 010010, China; sibinfeng@163.com (S.F.); tianchunyu@caas.cn (C.T.); yangyanting@caas.cn (Y.Y.); gongwenlong@caas.cn (W.G.); 2 Key Laboratory of Grassland Resources and Utilization of Ministry of Agriculture, Hohhot 010010, China

**Keywords:** *Bromus inermis*, mitochondrial genome, gene transfer, RNA editing, phylogenetic analysis

## Abstract

**Background**: *Bromus inermis* is a high-quality perennial forage grass in the Poaceae family, with significant ecological and economic value. While its chloroplast genome has been sequenced, the mitochondrial genome of this species remains poorly understood due to the inherent complexity and frequent recombination of plant mitochondrial genomes. **Methods**: We sequenced the complete mitochondrial genome of *B. inermis* using both Illumina Novaseq6000 and Oxford Nanopore PromethION platforms. Subsequently, comprehensive bioinformatics analyses were performed, including genome assembly and annotation, repetitive sequence identification, codon usage analysis, RNA editing site prediction, the detection of chloroplast-derived sequences, and phylogenetic reconstruction. **Results**: The mitochondrial genome of *B. inermis* was determined to be 515,056 bp in length, with a GC content of 44.34%, similar to other Poaceae species. This genome encodes 35 protein-coding genes, 22 tRNA genes, and 10 rRNA genes. Repetitive sequences account for 16.2% of the genome, totaling 83,528 bp, including 124 simple sequence repeats, 293 dispersed repeats, and 31 tandem repeats. A total of 460 RNA editing sites were identified, among which 430 were nonsynonymous. Additionally, 110 putative chloroplast-derived sequences were detected. A phylogenetic analysis based on mitochondrial genome data clarified the species’ evolutionary position within Poaceae. **Conclusions**: This study provides genetic resources for evolutionary research on and the communication of organelle genomes. Meanwhile, it also lays a solid foundation for the better development and utilization of the germplasm resources of *B. inermis*.

## 1. Introduction

*Bromus inermis* belongs to the genus *Bromus* in the family Poaceae, and it is a perennial herb with great ecological and economic value. It is native to Europe and widely distributed in Northeast China, North China, and Northwest China. It often grows naturally in diverse habitats, such as beside valley streams, wetlands along riversides, on both sides of roads, and at the edges of woodlands. It is rich in nutritional value, has a well-developed root system and strong stress resistance, and is highly regarded in fields such as animal husbandry and ecological restoration [1,2,3].

In eukaryotic cells, mitochondria are organelles that are encircled by a double layer of highly specialized membrane units. They serve as the energy source for the cell and are responsible for converting protein, fat, and glucose into ATP through aerobic respiration processes to power various cell functions [4,5]. The mitochondrial genome originated from the endosymbiotic event of *Alphaproteobacteria* 1.5 billion years ago and rapidly evolved through structural variation, rearrangement, and gene transfer [6,7]. While animal and algal mitochondrial genomes are generally compact and stable, plant mitochondrial genomes show far greater variability in size, sequence composition, and gene arrangement [8,9]. Moreover, plant mitochondrial genomes exhibit unique features in terms of gene transfer, transcription, and RNA processing [10,11,12]. The mitochondrial genomes of plants also exhibit considerable variation, even among closely related species [13].

Moreover, the size of angiosperm mitochondrial genomes can vary by up to an order of magnitude. For instance, the *Siberian larch* has the largest known mitochondrial genome at 11.7 Mb [14], while *Viscum scurruloideum* possesses the smallest, measuring only 66 kb [15]. In plants and animals, mitochondrial genomes vary greatly in structure and size, with the animal mitochondrial genome being relatively small, at approximately 16 kb, compared to 200–2000 kb in plants [16]. This variation is primarily attributed to repetitive sequences, gene transfer, and the acquisition or loss of genomic fragments [17,18]. The plant mitochondrial genome typically exists as a double-stranded circular structure, but linear, branched, and multichromosomal structures have also been observed [7,19]. Among these factors, the presence of abundant repetitive sequences not only increases the structural complexity of the mitochondrial genome, but also leads to genomic rearrangements through homologous recombination. These rearrangements can generate novel chimeric genes, which have been identified as causes of cytoplasmic male sterility [20,21]. The complex structure of mitochondrial genomes makes their sequencing and assembly more challenging than in other organellar genomes. Oda et al. completed the determination of the first plant mitochondrial genome in 1992 [22]. With advances in high-throughput sequencing technologies and assembly methodologies, an increasing number of plant mitochondrial genomes have been successfully assembled.

As of 1 March 2025, the GenBank database of the National Center for Biotechnology Information (NCBI) in the United States has included 78 complete mitochondrial genomes of Poaceae plants. Examples include *Elymus magellanicus* [23], *Triticum aestivum* [24], *Setaria italica* [25], *Hordeum vulgare* [26], and so on. A large proportion of them are in a circular structure, with the length ranging from 144,733 bp to 739,719 bp. Previous studies on *B. inermis* have mainly focused on genetic diversity analysis, cultivation management, and stress responses to adversity [27,28,29,30,31]. Although the chloroplast genome of *B. inermis* [32] has been assembled in previous studies, and its chloroplast genome sequence has been compared with those of species such as *Bromus biebersteini* [33] and *Bromus vulgaris*, currently, there is a lack of insights into the genetic evolution of *B. inermis* from the perspective of the mitochondrial genome. Assembling the mitochondrial genome of *B. inermis* is a prerequisite for a deeper understanding of its genetic characteristics, conducting molecular identification, and determining its taxonomic position within the Poaceae family. In this study, the mitochondrial genome of *B. inermis* was sequenced using the Novaseq6000 and PromethION sequencing systems. Subsequently, through a series of bioinformatics tools, genome annotation, repetitive sequence analysis, codon usage bias research, RNA editing site prediction, identification of chloroplast-derived sequences, and phylogenetic analysis were carried out. This study enriches the genetic information on and molecular markers of *B. inermis*, providing important references for its genomics research.

## 2. Materials and Methods

### 2.1. Mitochondrial DNA Extraction, Sequencing, and Assembly in B. inermis

The fresh leaves of *B. inermis* were collected in Hohhot, Inner Mongolia, China (40.57° N, 111.93° E). Professor Zinian Wu conducted a detailed identification of the plant material. The seeds (accession number: CF026121) and voucher specimens are stored in the National Perennial Forage Germplasm Resource Nursery (Hohhot, China). The genomic DNA was isolated and cleaned following the CTAB protocol [34], along with the Qiagen Blood & Cell Culture DNA Kit (Product No. 13323, Hilden, Germany). Sequencing was carried out using the Novaseq6000 (Illumina, San Diego, CA, USA) (BaseSum 40,609,024,742; ReadSum 135,045,374; GC (%) 45.25; Q20 (%) 97.66; Q30 (%) 93.43; average sequencing depth: 109.87×.) and PromethION (Oxford Nanopore Technologies, Oxford, UK) sequencing system (BaseSum 18,990,655,037; ReadSum 2,402,768; mean read length N50 7903, read length 19,866; average sequencing depth: 62.47×).

We employed minimap2 v2.1 [35] to align the PromethION (Oxford, UK) sequencing data against the reference gene sequences [36], thereby obtaining mitochondrial genome reads. Canu v2.2 [37] was used for read correction and trimming. Second-generation sequencing data were aligned to the calibrated reference sequence with Bowtie2 v2.3.5.1 [38]. Subsequently, the calibrated third-generation and aligned second-generation sequencing data were assembled using Unicycler v 0.4.8 [39] with default parameters. Finally, Bandage v 0.8.1 [40] was used to visualize and manually adjust the splicing results. The complete mitochondrial genome of *B. inermis* was assembled and deposited in GenBank (accession number: PQ510806; Supplementary Table S1). We used Bowtie2 v2.3.5.1 [38] to map the cleaned reads against the assembled mitochondrial genome to verify the accuracy of the assembly (Figures S1 and S2). Subsequently, SAMtools v1.17 [41] was employed to calculate the average coverage depth, and the coverage distribution was visualized using the Integrative Genomics Viewer (IGV) 2.17.3 [42].

### 2.2. Genome Annotation

Based on published mitochondrial genome data of Poaceaeplants, we annotated the *B. inermis* mitochondrial genome using GeSeq v2.05 [43] and PMGA [44]. Transfer RNA genes were predicted by tRNAscan-SE v2.0.12 [45] and optimized through manual validation to refine annotation results, ultimately constructing a complete circular mitochondrial genome model. Pseudogenes were identified based on GeSeq predictions and further confirmed by the following characteristics: (1) premature stop codons, (2) frameshift mutations, or (3) disruption of critical functional regions caused by insertions/deletions. In this study, the second-generation sequencing data of homologous materials were used to complete the assembly of the chloroplast genome through GetOrganelle v 1.7.0 [46]. The assembled chloroplast genome was annotated by the PGA v1.2.3 tool [47], and the organelle genome mapping software OGDRAW v1.3.1 [48] was employed to visualize the genome map.

### 2.3. Repeat Sequence Identification

The assembled mitochondrial genome was analyzed for simple sequence repeats (SSRs) using MISA v 1.0 [49]. The minimum numbers of monomers, dimers, trimers, tetramers, pentamers, and hexamers were set to 10, 5, 4, 3, 3, and 3, respectively. The default parameters of a Tandem Repeat Finder v 4.09 [50] were utilized to detect tandem repetitions. REPuter v 2.74 [51] was utilized to identify dispersed repeats ≥30 bp in length, with classification based on a Hamming distance threshold of 3 and an E-value cutoff of 1 × 10^−5^, categorizing them as forward, reverse, palindromic, or complementary repeats. Among them, setting the E-value at 1 × 10^−5^ can effectively identify reliable dispersed repeats while minimizing misjudgment results.

### 2.4. Codon Preference Patterns in B. inermis

Molecular evolutionary genetics software MEGA v 11.0.26 [52] was used to determine the relative synonymous codon usage (RSCU) value of protein-coding genes (PCGs) and the amino acid composition. Perl scripts were used to configure codon preference.

### 2.5. Prediction of RNA-Editing Sites

The prediction of C-to-U RNA editing sites in mitochondrial genomes was performed using Deepred-Mt v 1.0.0 [53], a convolutional neural network (CNN)-powered tool. The prediction process involved extracting all mitochondrial protein-coding genes, which were then analyzed with Deepred-Mt. We consider the prediction results with a probability value exceeding 0.9 to be reliable because a relatively high threshold significantly reduces false positive results.

### 2.6. Identification of the Mitochondrial Sequence Obtained from Chloroplasts

We acquired the mitochondrial genome information from our assembly. It was discovered that the genomes of mitochondria and chloroplasts contain homologous segments. BLASTN was used to screen the transmitted DNA fragments, with the E-value threshold set at 1 × 10^−5^. Gene translocation from chloroplasts to mitochondria was analyzed with TBtools v 2.091 [54].

### 2.7. Phylogenetic Analysis

The evolutionary analysis utilized a set of 25 essential PCGs for phylogenetic tree construction. The full mitochondrial genomes of 33 Poaceae species were used to create these PCGs, with *Glycine max* and *Lotus japonicus* serving as outgroups. The mitochondrial genome reference sequences were acquired from the NCBI database (Supplementary Table S1). The concatenated PCGs nucleotide sequences from the selected genome were aligned using MAFFT v 7.131 [55] for multiple sequence analysis. Using Bayesian inference (BI) techniques and Maximum Likelihood (ML) algorithms, a phylogenetic tree was created, and ModelFinder v1.1 was used to choose the best model [56]. The ML approach computes the GTRGAMMAI model with 1000 duplicate bootstraps using RAxML [57]. The Bayesian phylogenetic tree was reconstructed using MrBayes v3.2.6 with the optimal substitution model GTR+F+I+G4 [58].

## 3. Results

### 3.1. Mitochondrial Genome Annotation and Genomic Analysis of B. inermis

**Figure 1:**
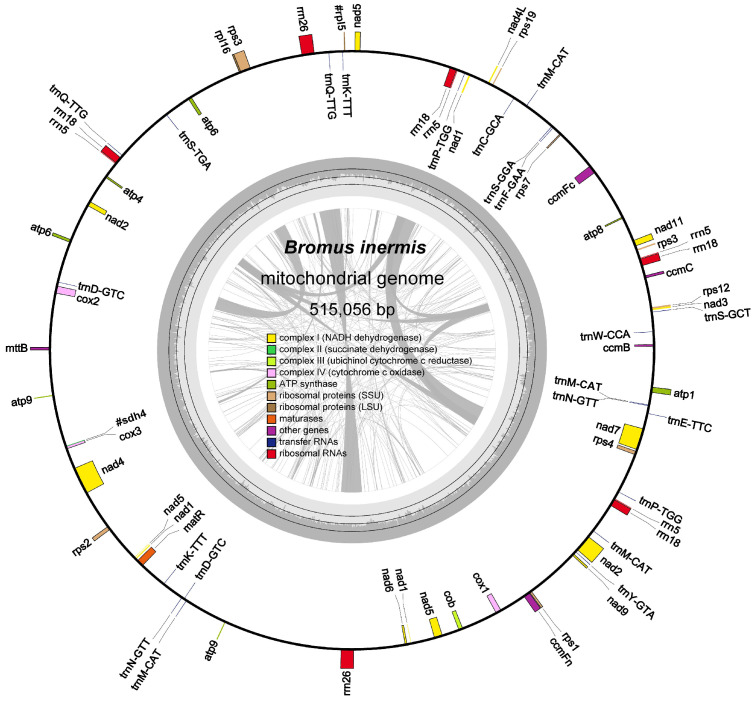
The entire mitogenome of *Bromus inermis* represented as a circle. The forward-transcribed and reverse-transcribed genes are those that are situated outside and within the circle, respectively. The GC content is shown by carbon gray region of the inner circle. Genes with the same function are shown in the same color, whereas various functional gene groups are coded in separate colors.

The mitochondrial genome of *B. inermis* was assembled and found to exhibit a circular conformation, a common feature among Poaceae species. The mitochondrial genome of *B. inermis* displays characteristic features with a total length of 515,056 bp and a GC composition of 44.34%. The genomic annotation identified 35 PCGsthat collectively span 31,590 bp, constituting 61.3% of the entire mitogenome. The assembly further revealed the presence of 10 ribosomal RNA genes totaling 15,316 bp (2.97%) and 22 transfer RNA genes encompassing 1668 bp (0.32%) (Supplementary Table S2, Figure 1).

The functional annotation of the mitochondrial genome revealed two distinct gene categories. The core functional genes comprise three cytochrome c oxidase subunits (*cox1*, *cox2*, *cox3*), four cytochrome c biogenesis factors (*ccmB*, *ccmC*, *ccmFc*, *ccmFn*), five ATP synthase subunits (*atp1*, *atp4*, *atp6*, *atp8*, *atp9*), and one maturase enzyme (*matR*). The accessory gene repertoire includes nine NADH dehydrogenase subunits (*nad1*-*nad7*, *nad9*, *nad4L*), eight small ribosomal proteins (*rps1*-*rps4*, *rps7*, *rps12*, *rps13*, *rps19*), one membrane transport protein (*mttB*), and one large ribosomal protein subunit (*rpl16*). In addition, two pseudogenes similar to *rpl5* and one pseudogene similar to *sdh4* were also detected (Supplementary Table S2).

Most genes exist in the form of single copies. However, gene duplication was observed in the ATP synthase family, with both *atp6* and *atp9* being present as two distinct copies within the mitochondrial genome. Among the three types of rRNA genes, *rrn26* is present in two copies, whereas *rrn18* and *rrn5* each occur in four copies. In the case of the 14 tRNA genes, *trnD-GTC*, *trnk-TTT*, *trnN-GTT*, *trnP-TGG*, and *trnQ-TTG* are each present in two copies, while *trnM-CAT* exists in four copies (Supplementary Table S2). A comparative analysis of the mitochondrial coding genes revealed substantial size variation, with the *nad5* gene being the largest (2,013 bp), encoding 671 amino acids, while the *atp9* gene was the smallest (225 bp), encoding only 75 amino acids (Supplementary Table S3). Among these 57 genes, both *ccmFc* and *cox2* contain one intron region each. Among the NADH dehydrogenase genes, *nad1*, *nad2*, *nad5*, and *nad7* each contain four introns, whereas *nad4* contains three introns. Additionally, among the ribosomal protein genes, both *rps1* and *rps3* possess one intron each (Supplementary Table S4).

### 3.2. Mitochondrial Genome Repeats Sequence Analysis

**Figure 2:**
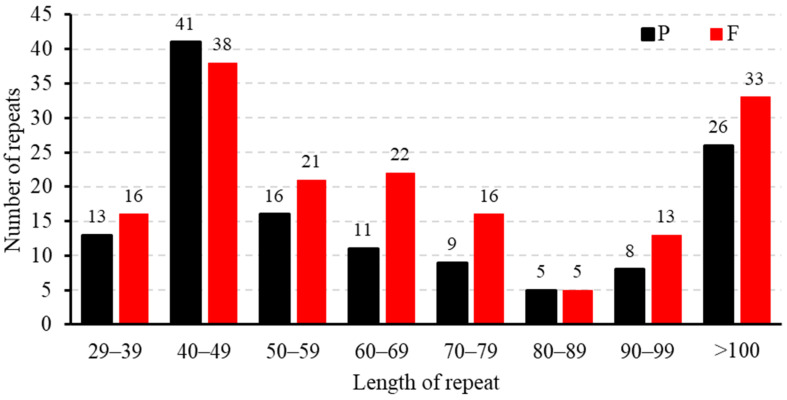
Dispersed repeat length distribution in the mitochondrial genome of *B. inermis*. The number of distributed repetitions is shown by the ordinate, the type of dispersed repeats is represented by the *x*-axis, and the length of the repeats changes according to the type.

The findings of this study align with the repetitive sequence types observed in other gramineous plants, predominantly comprising tandem repeats, dispersed repeats, and SSRs. However, there are differences in the number of repetitive sequences. The repeat sequence analysis of the *B. inermis* mitogenome identified 293 interspersed repetitive elements, distributed as 56% palindromic (164) and 44% forward (129) repeats. Neither reverse nor complementary repeat sequences were detected in the assembly (Figure 2). In the study of repetitive sequence lengths, we observed that 80% of the repetitive sequences were in the range of 29 to 100 bp, while the remaining 20% exceeded 100 bp. Notably, seven repetitive sequences have lengths exceeding 1 kb, and two sequences even surpass 10 kb, which is closely related to homologous recombination (Supplementary Table S5).

**Figure 3:**
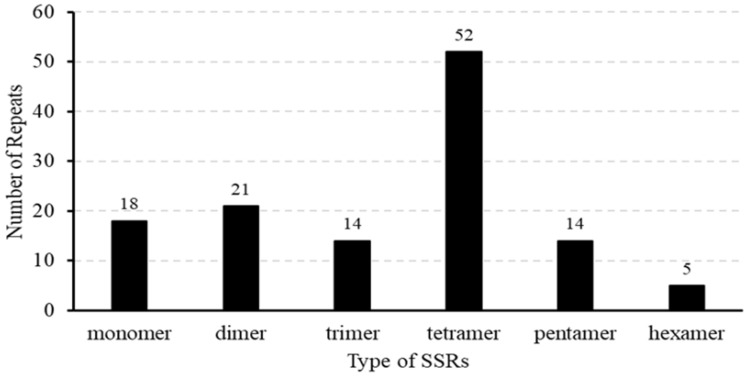
SSRs are distributed throughout the mitochondrial genome of *B. inermis*. The number of SSRs is represented by the ordinate, whereas the repeat type of SSR is represented by the *x*-axis.

**Figure 4:**
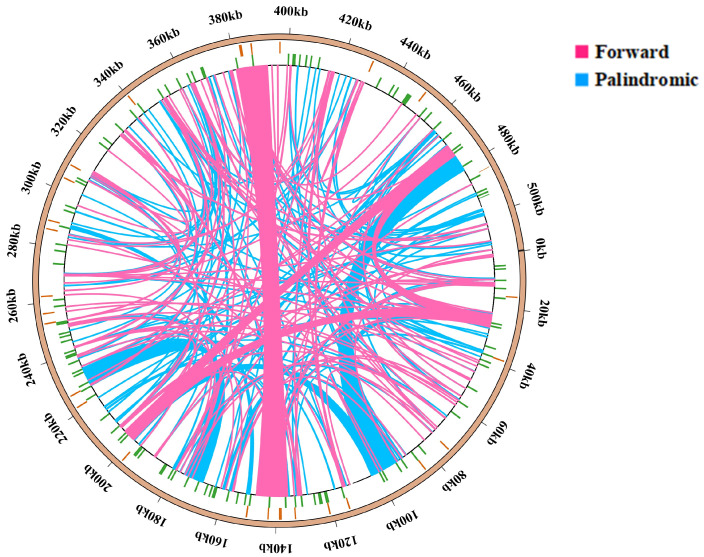
Repetitive sequence distribution in the mitochondrial genome of *B. inermis*. The complete genomic sequence is represented by the outermost circle, tandem repeats (orange), simple sequence repeats (green), scattered repeats, and mitochondrial genome sequences are represented by circles, going from the outside to the inside. Notably, 129 forward repeats and 164 palindromic repeats are visualized as pink and blue arcs, respectively.

We also detected 124 SSRs in the mitochondrial genome of *B. inermis*. Among them, there were 18 mononucleotide sequences (14%), 21 dinucleotide repeat sequences (16%), 14 trinucleotide repeat sequences (11%), 52 tetranucleotide repeat sequences (41%), 14 pentanucleotide repeat sequences (11%), and five hexanucleotide repeat sequences (4%) (Supplementary Table S6, Figure 3). Among all the SSRs, over half of the repetitive sequences are A/T-rich. Notably, 33 SSRs are entirely composed of A/T, comprising 15 monomers (A/T), 12 dimers (AT/TA), three trimers (TAT, TTA, TAA), and three tetramers (ATAA, TTAT, AATA) (Figure 3). The A/T richness of 42 SSRs varies between 40% and 84%. Additionally, 115 SSRs are located in the IGS region, with two SSRs each located on the introns of *nad4*, *nad2*, and *nad7*, and none are distributed on the exons. Furthermore, two SSRs are distributed in the open reading frame (ORF) region (Supplementary Table S7). The mitochondrial genome assembly of *B. inermis* revealed the presence of 31 tandem repeat elements. These repeat sequences are evenly distributed, with lengths ranging from 4 to 81 bp, and the similarity match is greater than 78%. Moreover, 30 of the tandem repeat sequences are located in the IGS region, and one is located in the *rps2* gene (Supplementary Table S8). The mitochondrial genome of *B. inermis* is rich in repetitive sequences, comprising 124 SSRs, 31 tandem repeats, and 293 dispersed repeats. With a total length of 83,528 bp, these repeats account for 16.2% of the genome’s total length, reflecting a remarkably high density of repetitive sequences in this mitochondrial genome (Figure 4).

### 3.3. PCGs Codon Bias Analysis

**Figure 5:**
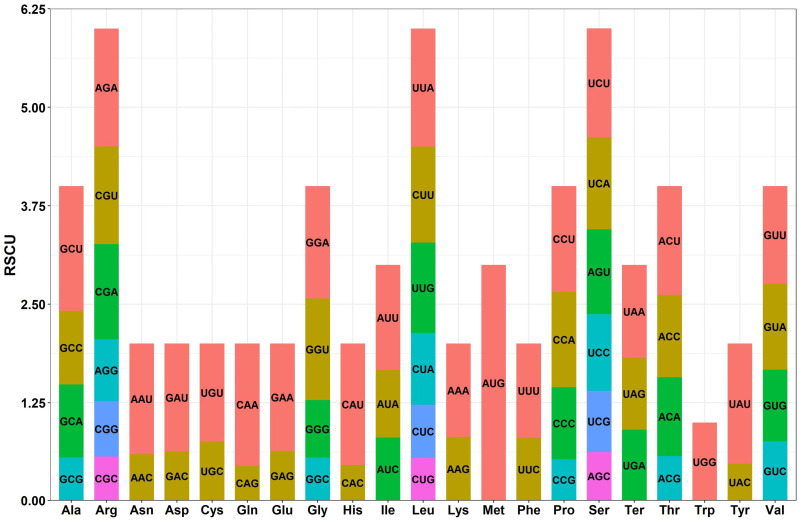
Protein-coding codon usage patterns in *B. inermis*. The vertical axis shows the relative synonymous codon usage (RSCU) value, whereas the horizontal axis shows the codon family.

The mitochondrial genome of *B. inermis* exhibits distinct codon usage patterns among its 35 protein-coding genes. While most genes (33/35) initiate translation with the standard ATG start codon, two exceptions (*nad1* and *nad4L*) utilize ACG instead. The mitochondrial genome of *B. inermis* exhibits four distinct termination codon patterns in its protein-coding genes. TAA serves as the stop codon for 13 genes, including *atp8*, *cox2*, multiple NADH dehydrogenase genes (*nad1*, *nad2*, *nad3*, *nad4L*, *nad5*, *nad6*, *nad9*), and ribosomal protein genes (*rpl16*, *rps4*, *rps7*, *rps19*). TGA functions as the termination codon in 11 genes, notably ATP synthase genes (*atp1*, both copies of *atp6* and *atp9*), cytochrome c biogenesis genes (*ccmB*), *cox3*, *nad4*, and ribosomal protein genes (*rps1*, *rps12*, *rps13*). TAG acts as the stop codon for 10 genes, including *atp4*, cytochrome c biogenesis genes (*ccmC*, *ccmFn*), *cob*, *cox1*, maturase genes (*matR*), membrane transport protein genes (*mttB*), *nad7*, and ribosomal protein genes (*rps2*, *rps3*). Notably, *ccmFc* uniquely utilizes CGA as its termination codon (Supplementary Table S3). The PCGs have a length of 31,590 bp and encode 10,174 codons. Among thePCGs, leucine (Leu) is the most common amino acid, occurring 1097 times (accounting for 10.78%). It is followed by serine (Ser, accounting for 8.10%), arginine (Arg, accounting for 6.74%), and alanine (Ala, accounting for 6.47%). In contrast, tryptophan (Trp) and methionine (Met) are the least common. Additionally, there are 30 types of codons with a RSCU value greater than 1. The RSCU value of AUG for methionine is 3.0, indicating its predominant usage as the codon for this amino acid. For arginine, the RSCU value of CGG is one, indicating that there is no preference for using this codon. Moreover, In *B. inermis*, the most frequently used synonymous codons show a clear base preference: A/T bases are more prevalent than G/C bases at the third codon position. This pattern aligns with the codon composition observed in most plant mitochondrial genomes (Supplementary Table S9, Figure 5).

### 3.4. Identification of RNA-Editing Sites in Genes That Code for Proteins

**Figure 6:**
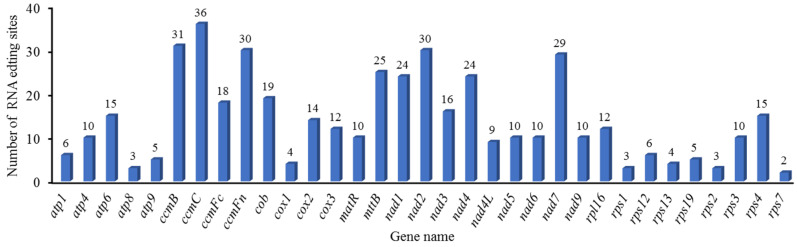
Distribution Patterns of RNA Editing Sites in *B. inermis* mitochondrial PCGs.

**Figure 7:**
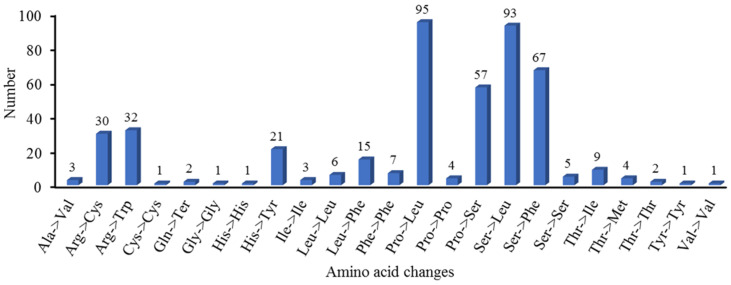
Amino acid changes in *B. inermis* mitochondrial PCGs.

RNA editing represents an important post-transcriptional modification mechanism that can modify mRNA coding sequences and, consequently, impact protein structure and function. Our analysis identified 460 high-confidence RNA editing sites (prediction probability >90%) distributed across 35 PCGs in the mitochondrial genome. The stringent prediction threshold significantly enhances the reliability of these results by minimizing false-positive identifications. Furthermore, all the base changes at these sites were from C to U. Among the RNA editing sites, the NADH dehydrogenase gene harbored the highest number (162), accounting for 35.2%. Specifically, *nad1* had 24, *nad2* had 30, *nad3* had 16, *nad4* had 24, *nad4L* had nine, *nad5* had ten, *nad6* had ten, *nad7* had 29, and *nad9* had 10. The gene related to cytochrome c biosynthesis had the second largest number of RNA editing sites, with 115, accounting for 25%. Specifically, *ccmB* had 31, *ccmC* had 36, *ccmFc* had 18, and *ccmFn* had 30. The ribosomal protein (small subunit) gene contained 48 RNA editing sites, accounting for 10.43%. Specifically, *rsp1* had three, *rps12* had six, *rps13* had four, *rps19* had five, *rps2* had three, *rps3* had ten, *rps4* had 15, and *rps7* had two. Other genes contained relatively few RNA editing sites (Supplementary Table S10, Figure 6). Among the 460 RNA editing sites, 430 are non-synonymous editing sites and 30 are synonymous editing sites. Editing primarily occurs at the second base of the codon (271 cases, 58.9%), followed by the first base (161 cases, 35%), with only 28 cases (6.1%) at the third base. Additionally, 23 forms of amino acid changes are observed. The most common amino acid change is proline to leucine, which occurs 95 times, followed by serine to leucine, serine to phenylalanine, and proline to serine, occurring 93, 67, and 57 times, respectively. Most of these transitions are towards more hydrophobic amino acids. Moreover, changes such as proline to leucine and proline to serine may affect the stability and continuity of α-helices, thereby altering the secondary structure of proteins (Supplementary Table S10, Figure 7).

### 3.5. Mitogenomic Sequences Obtained from Chloroplasts

**Figure 8:**
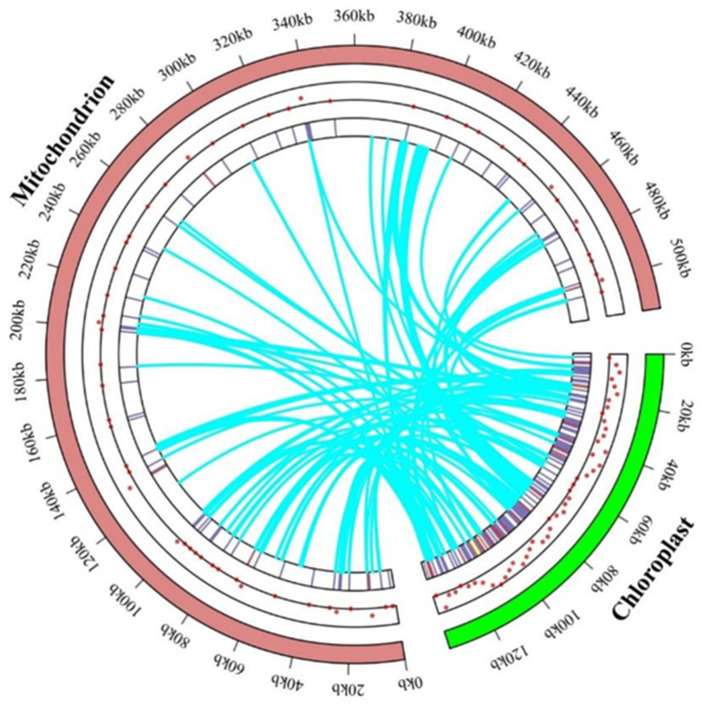
Events occurring that transfer genes between the genomes of mitochondrion and chloroplast. The locations of migratory genes inside the two chromosomes are indicated by dots and heat maps. The mitochondrial genome is represented by the brick color part, and the chloroplast genome is represented by the green spherical part. The process by which chloroplast-like sequences are transferred from the chloroplast genome into the mitochondrial genome is shown by the curved blue line in the circle.

The complete mitochondrial genome of *B. inermis* is 515,056 bp in length, approximately 4.47 times the size of its chloroplast genome (115,100 bp) (Figure 8). To reliably identify sequence similarities while excluding random short fragment matches, we employed BLASTN with strict E-value parameters for mitochondrial–chloroplast genome alignment in *B. inermis*. We identified 110 chloroplast fragments in the mitochondrial genome that may have undergone gene transfer, with their similarities ranging from 67.7% to 100%. The total length of these inserted fragments is 33,065 bp, comprising 6.41% of the mitochondrial genome’s total length. Transferred sequences predominantly migrate from the chloroplast genome’s PCGs, tRNA genes, and IGS region to the mitochondrial genome’s rRNA genes and IGS region. Among these, the longest sequence (5539 bp) is transferred from the *psaA*, *rps14*, and *psaB* genes of the chloroplast genome to the intron region of *nad2* in the mitochondrial genome. Among the remaining transferred sequences, 38 are situated in the mitochondrial genome’s IGS region and have lost functionality, while 32 reside on *rrn18*, 20 on *rrn26*, one on *atp1*, two on *rps12*, one on *nad5*, and 16 on tRNA, all maintaining functional integrity (Supplementary Table S11).

### 3.6. Phylogenetic Analysis

**Figure 9:**
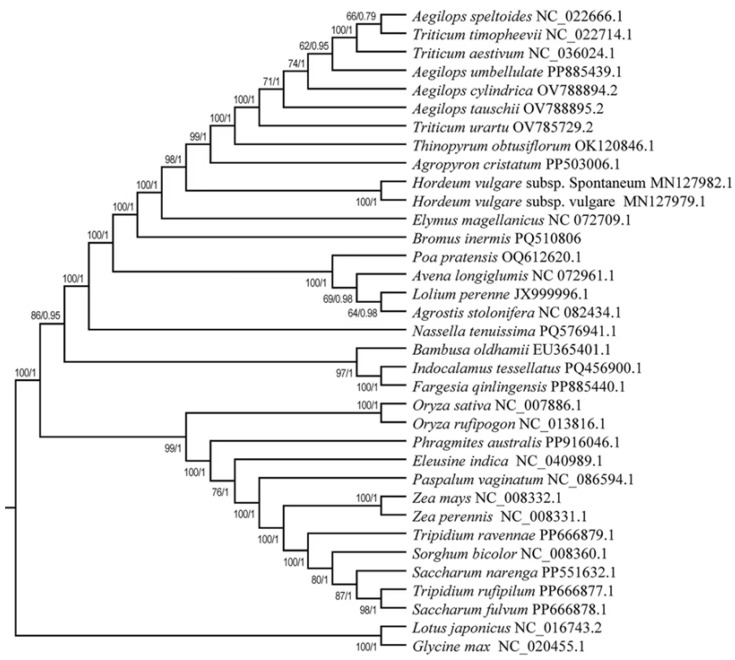
Phylogenetic relationships between 32 Poaceae species and *B. inermis*. The ML bootstrap support values/BI posterior probabilities are shown for each node.

In order to study the evolutionary relationships of the mitochondrial genome of *B. inermis*, a phylogenetic tree of *B. inermis* and 32 other Poaceae plants was constructed by using the ML and the BI. According to the evolutionary relationships of the mitochondrial genomes within the taxonomic group, the taxonomic group was divided into three distinct branches (Figure 9). Among all the species, besides *Aegilops speltoides* along with *Triticum timopheevii*, *Hordeum vulgare* subsp. Spontaneum along with *Hordeum vulgare* subsp. vulgare, *Lolium perenne* along with *Aegilops speltoides*, *Indocalamus tessellatus* along with *Fargesia qinlingensis*, *Oryza sativa* along with *Oryza rufipogon*, *Zea mays* along with *Zea perennis*, and *Tripidium rufipilum* along with *Saccharum fulvum* formed the dependent clade. Other species all formed the individual clade by itself. Among the 33 nodes of this phylogenetic tree, 32 nodes have a support rate exceeding 90%, and 18 nodes have a support rate reaching 100%. Additionally, 28 nodes have a posterior probability of 1, four nodes have a posterior probability above 0.9, and one node has a posterior probability of 0.79. This demonstrates the reliability of our research findings. In terms of the *B. inermis* that we focused on, it has formed a separate clade by itself, and it has a closer relationship with *E. magellanicus*, two subspecies of *Hordeum* and other four species subtribed to *Aegilops*, three *Triticum* species, *Thinopyrum obtusiflorum*, and *Agropyron cristatum*.

## 4. Discussion

### 4.1. Structural Characteristics of the Mitogenome

Compared to animals, plant mitochondrial genomes are generally larger and more structurally diverse, including circular, linear, branched, and mixed forms, with circular mitochondrial genomes comprising the majority [59]. In this study, the mitochondrial genome of *B. inermis* was assembled into a single circular structure with a length of 515,056 bp, which is not remarkable compared to other Poaceae plants (Supplementary Table S1). However, its GC content and the number of mitochondrial genes are relatively consistent with those of *A. cristatum* [60], *Avena longiglumis* [61], *S*. *italica* [25], *Poa pratensis* [62], and *T. obtusiflorum* [63].

The mitochondrial genome structure of *B. inermis* is similar to those of published gramineous plants, mostly exhibiting a circular structure, which is partly attributed to the low occurrence of homologous recombination and the conserved structure [64]. However, the mitochondrial genomes of some Poaceae plants show multi-circular, linear, or complex branched structures, such as maize [65] and sugarcane [66]. Due to the lack of promoter or the phenomenon of premature stop codon or frameshift mutations, the functionless paralogs generated from ancestral functional genes were the pseudogenes [67]. In this research, two pseudogenes, *rpl5* and *sdh4*, were annotated. Because of the absence of these two genes in the chloroplast genome of *B. inermis* [68], we speculated that pseudogenization might also be produced by genes’ transformation from chloroplasts to mitochondrial genomes, which were the similar with *melastoma* [69]. However, this presumption needs to be proven further.

### 4.2. Repeated Sequences of the Mitochondrial Genome

Repetitive sequences, including SSRs, tandem repeats, and dispersed repeats, can alter genome size through structural variations and increase the diversity of mitochondrial genomes [70]. This is part of the reason for the size differences in plant mitochondrial genomes. The main cause can be attributed to variations in intergenic regions [71]. Overall, frequent gene gain, loss, transfer, replication, and genome rearrangement events in mitochondrial DNA lead to huge differences in genome sizes among different species [72]. Due to the characteristics of high polymorphism and rich content among different species, SSRs can be selected for the development of molecular markers, identification of genetic resources, and phylogenetic analysis. In this study, 124 SSRs are dispersed in different gene regions. The most common SSRs are tetranucleotide repeat sequences, accounting for 41.9%. Moreover, all SSRs are composed of motifs that are rich in A and T. In *T. obtusiflorum*, there is also an A+T bias (55.61%), which confirms the correlation between the AT content of the complete mitochondrial genome and the SSRs [63].

In addition, the presence of numerous repetitive sequences can lead to structural rearrangements of the genome. Large repetitive sequences are active in intramolecular recombination, such as in *Brassica napus* [73]. It is worth noting that the occurrence of super-large repetitive sequences mediates more active homologous recombination [74,75].

For example, in *Gossypium raimondii* [76], *Arabidopsis* [77], and sugar beet [78], ultra-long repetitive sequences exhibit active performance in homologous recombination. For circular chromosomes, when the repeats are present in opposite directions, circular isomeric forms will be formed. When the repeats are present in a direct orientation, two small circular genomes (subgenomes) will be formed [13].

In *B. inermis*, these extremely long repeat sequences are highly likely to mediate homologous recombination, and seven repeat sequences with a length exceeding 1000 bp may also be involved. Among the 293 dispersed sequences, there are 129 direct repeat sequences (F; 44%), which indicates that homologous duplication mediated by repeat sequences is highly likely to form subgenomes. There are 164 palindromic repeat sequences (P; 56%). When homologous recombination occurs, the order of gene arrangement may change accordingly, further forming cyclic isomers with different conformations.

### 4.3. The Preference of Amino Acids for Codons

Codon bias refers to the phenomenon in which the usage frequencies of different codons encoding the same amino acid vary in organisms. This bias is of great significance in the processes of genetic information transfer and protein synthesis in living organisms [79]. Among the 35 PCGs in the mitochondrial genome of *B. inermis*, leucine is the amino acid with the highest frequency of occurrence.

Since the three codons, AUU, AUC, and AUA, all encode leucine, base mutations may not change the type of amino acid. Therefore, the degeneracy of codons reduces the impact of gene mutations on protein structure and function, ensuring the accuracy and stability of protein synthesis. Furthermore, leucine has a large hydrophobic side chain, which means that it tends to aggregate inside the protein during protein folding, helping to maintain the three-dimensional structure of the protein [80]. To some extent, this is conducive to the stable transmission of biological genetic information. For example, in many membrane proteins, leucine-rich regions often participate in the formation of transmembrane domains, stabilizing the localization of proteins in the cell membrane [81].

Synonymous codon usage exhibits significant biases across different species. In most plants, codons ending with A/T at the third base are more prevalent, suggesting that the codon preference patterns among genomes may be associated with A/T content bias. In contrast, variations in codon usage among different genes within the same genome are likely influenced by tRNA abundance [82,83,84,85].

From the perspective of codon usage, the analysis of the RSCU values shows that the RSCU values of some codons are greater than one, indicating that these codons are preferentially used when encoding the corresponding amino acids. For example, the AUG codon encoding methionine has an RSCU value as high as three, making it one of the codons with the highest usage frequency. The RSCU value of the CGG codon for arginine is one, indicating that arginine has no obvious preference for this codon. Arginine has multiple codons (such as CGU, CGC, CGA, CGG, AGA, and AGG). This diversity in codon usage may provide certain evolutionary advantages for organisms [86].

### 4.4. RNA-Editing-Related Amino Acid Alterations

RNA editing sites are specific nucleotide sites in an RNA molecule that can be changed, such as through base additions, deletions, or transfer, resulting in RNA sequences that differ from the original DNA template sequences. This editing process can alter RNA coding information, affect protein structure and function, and regulate RNA stability and metabolism [87,88]. RNA editing plays an important role in organisms. RNA editing could increase protein diversity, correct RNA sequence errors, and regulate gene expression, as well as RNA metabolism. In general, there were more RNA editing sites in plant mitochondria than in chloroplasts [89]. The 460 RNA editing events in the mitochondrial genome of *B. inermis* are conversions from C to U. This change is the primary way in which plants modify their mitochondrial RNA, and it is crucial in controlling the expression of certain genes. Based on existing research, there were 491 RNA editing sites in *O. sativa* [90] and 441 RNA editing sites in *Arabidopsis thaliana*. However, only 81 RNA editing sites were found in the *A. cristatum* mitogenome. Moreover, the predominant RNA editing event is the C-to-T transition, which is consistent with previously reported editing characteristics [91]. Therefore, we concluded that the RNA editing events existed extensively among different species. Furthermore, *O. sativa*, *A. thaliana*, and *A. cristatum* may need more RNA editing acting as a buffer against less favored mutations in the genomic coding sequences [92,93,94]. But this presumption still needs to be improved. Compared with *Agrostis stolonifera* and diploid oat *A. longiglumis*, *B. inermis* had a similar RNA editing mode. For instance, the genes *nad2* and *nad7* both had more RNA editing sites in *B. inermis* and the two aforementioned species. Furthermore, *ccmC* and *ccmFn* also have more editing sites than other genes in the *B. inermis* and *A. stolonifera* mitogenomes. According to the relevant studies on wheat, for the correct expression of *nad2*, besides splicing processes, mRNA editing is also required [95]. Hence, we inferred that RNA editing was also necessary during the expression of *nad2* and *nad4* in the *B. inermis* mitogenome. But this assumption needs further verification. In *B. inermis*, RNA editing predominantly occurs in 35 PCGs, which can be classified into nine functional groups: ATP synthase, cytochrome c biogenesis, ubiquinol cytochrome c reductase, cytochrome c oxidase, maturases, transport membrane proteins, NADH dehydrogenase, ribosomal proteins (LSUs), and ribosomal proteins (SSUs). This phenomenon is consistent with most plants, in which no RNA editing occurs in rRNA and tRNA genes, further confirming the highly conserved nature of these two classes of RNA molecules during evolution. Their sequences and spatial structures remain relatively stable across different species [96].

### 4.5. Gene Transfer and Phylogenetic Evolution

In recent years, genomic studies have revealed frequent gene transfer events among different subcellular genomes (mitochondrial, nuclear, and chloroplast) in plants [97]. In angiosperms, frequent integration of chloroplast-derived sequences into mitochondrial genomes has been observed, with these transferred sequences constituting 1–12% of the total mitochondrial DNA length. This phenomenon appears to be significantly less common in non-angiospermous plants and green algae [6,98]. Studies have shown that the transfer of chloroplast DNA fragments to the mitochondrial genome is a common phenomenon during the evolution of higher plants, leading to the frequent presence of chloroplast DNA sequences in plant mitochondrial genomes. Moreover, the frequency of such transfers is positively correlated with the size of the mt genome [99,100]. Our research has found that there are 110 gene fragments in the mitochondrial genome of *B. inermis* that are likely to have been transferred from chloroplasts, accounting for 6.41% of the total length of the mitochondrial genome. In *A. cristatum*, 9% of the fragments derived from chloroplasts have been identified. In *Acer truncatum Bunge* [101], the proportion is 2.36%. The proportions in *Salix suchowensis* and *Suaeda glauca* are 2.8% and 5.18%, respectively [102]. This reflects that there are differences in the proportion of chloroplast-derived gene fragments in the mitochondrial genomes of different plants, and the phenomenon of gene fragment transfer shows diversity among different plant species. This may be related to various factors, such as the evolutionary history, physiological characteristics, and environmental adaptability of different plants. In addition, it is noticeable that this genetic migration induces changes in functional genes [103]. During the process of chloroplast gene transfer to mitochondria, multiple transfer types exist, including both gene transfers with completely preserved functions and structures, as well as different forms of transfers, ranging from non-coding regions to complete genes. In our study, we found that protein-coding genes and rRNA genes are prone to structural variations during transfer, while tRNA genes can often maintain functional stability. This phenomenon is consistent with the research results on *A. cristatum* (crested wheatgrass) and soybean [104].

Phylogenetic trees reveal their evolutionary history and taxonomic status by showing the relationships and evolutionary paths of different species of Poaceae [105]. In the study on the phylogenetic relationships of Poaceae, Zhang et al. constructed high-resolution phylogenetic trees of the Poaceae family through extensive sampling and sequencing, combined with multi-gene sequence analysis. This study not only supports the monophyletic nature of several subfamilies in Poaceae, but also provides an important theoretical basis for the classification system of Poaceae by newly analyzing the systematic relationships among families and subfamilies [106]. To elucidate the phylogenetic relationships, from the common species that had published mitogenomes that we could search for in the NCBI, subtribes of different genera of the Poaceae family were chosen to perform the analysis. The phylogenetic tree constructed by us shows the evolutionary relationships between *B. inermis* and various Poaceae plants. Along with the plants of the genera *Aegilops*, *Triticum*, *Thinopyrum*, *Agropyron*, *Hordeum*, *Elymus*, *Poa*, *Avena*, *Lolium*, *Agrostis*, *Nassella*, *Bambusa*, *Indocalamus*, and *Fargesia*, it belongs to the same major branch, which indicates that they have a relatively close genetic relationship in evolution and share a common ancestor, and that the divergence time is relatively recent. This result was partially consistent with the traditional taxonomy and other molecular biology studies, and provides strong evidence for the taxonomic status of *B. inermis* from the perspective of the mitochondrial genome.

## 5. Conclusions

In this study, the complete mitochondrial genome of *B. inermis* was successfully assembled and annotated. It is 515,056 base pairs in length, containing 57 genes and various repetitive sequences. We also conducted in-depth evaluations of gene transfer, RNA editing, and codon usage. As a result, 110 gene fragments potentially originating from the chloroplast were discovered, 460 RNA editing sites were detected, most of which were non-synonymous editing sites, and the codon usage bias was identified. By comparing the mitochondrial genomes of 32 Poaceae plants and constructing a phylogenetic tree, the origin and differentiation relationships of *B. inermis* and its related species were clarified. It has a close genetic relationship with plants from multiple genera. These findings reveal important mitochondrial genome information on *B. inermis*, providing valuable evidence for the full utilization of its germplasm resources and offering new evidence for research into its genetic evolution.

## Data Availability

The complete mitochondrial genome sequences of the *B inermis* we sequenced were deposited in the NCBI (https://www.ncbi.nlm.nih.gov/, accessed on 25 October 2024); the GenBank accession number is the following: PQ510806.

## Supplementary Materials

The following supporting information can be downloaded at https://www.mdpi.com/article/10.3390/genes16060652/s1, Figure S1: Quality and coverage of second-generation sequencing; Figure S2: Quality and coverage of third-generation sequencing; Table S1: Material information for phylogenetic tree analysis in this study; Table S2: The gene makeup of the mitogenome of *B. inermis*; Table S3: Mitochondrial gene start codon and stop codon and fragment information of *B. inermis*; Table S4: Intron and exon sizes and directions of dividing genes in the mitochondrial genome of *B. inermis*; Table S5: Size and type of scattered repeats in mitochondrial genome of *B. inermis*; Table S6: Distribution of SSRs types and quantities in mitochondrial genome of *B. inermis*; Table S7: SSRs in the mitochondrial genome of *B. inermis*; Table S8: Size and location distribution of tandem repeats in mitochondrial genome of *B. inermis*; Table S9: Codon counts in the genes that code for mitochondrial proteins in *B. inermis*; Table S10: The information table for *B. inermis* mitochondrial genomic RNA editing sites; ** indicates the acquisition of the start codon, * indicates the acquisition of the stop codon; Table S11: Size and location of fragments transferred from chloroplast genome to mitochondrial genome in *B. inermis*.

## Abbreviations

The following abbreviations are used in this manuscript:

NCBI	National Center for Biotechnology Information
tRNA	Transfer RNA
rRNA	Ribosomal RNA
PCGs	Protein-coding genes
ORF	Open Reading Frame
IGS	Intergenic spacer
SSRs	Simple sequence repeats
RSCU	Relative synonymous codon usage ratios
BI	Bayesian inference
ML	Maximum Likelihood
