# Supplementary material for: Assembly and Comparative Analysis of the Complete Mitochondrial Genome of Bromus inermis
Source: Genes (Basel). 2025 May 28;16(6):652. doi: 10.3390/genes16060652 (PMC12193292; doi:10.3390/genes16060652)
Supplement: Supplementary file 1 [file genes-16-00652-s001.zip › genes-3628464-supplementary Figure.pdf]

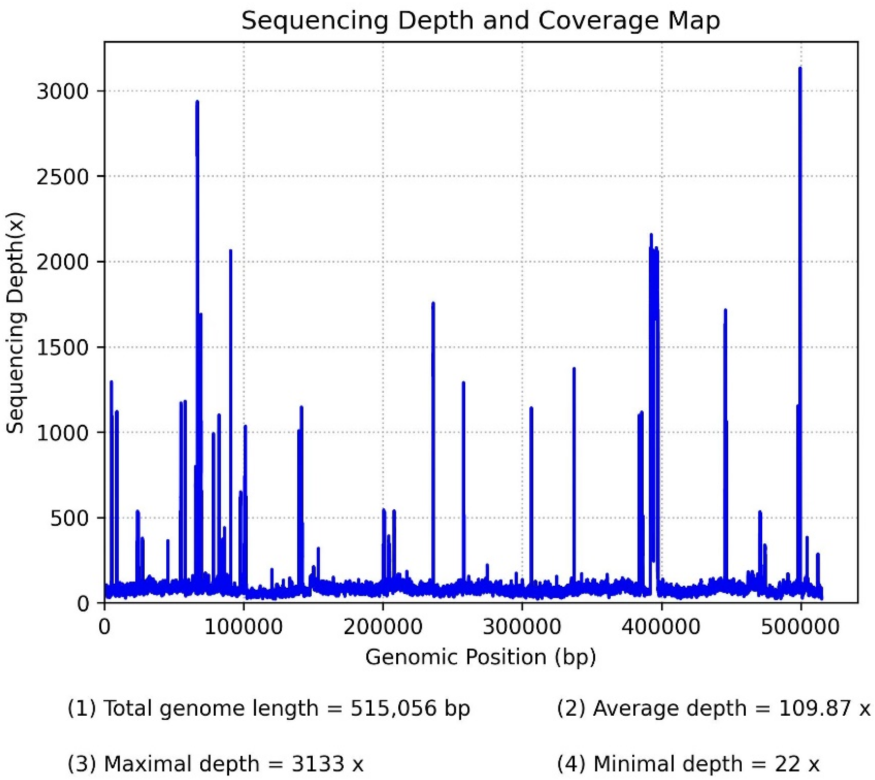

Figure S1. Quality and coverage of second - generation sequencing

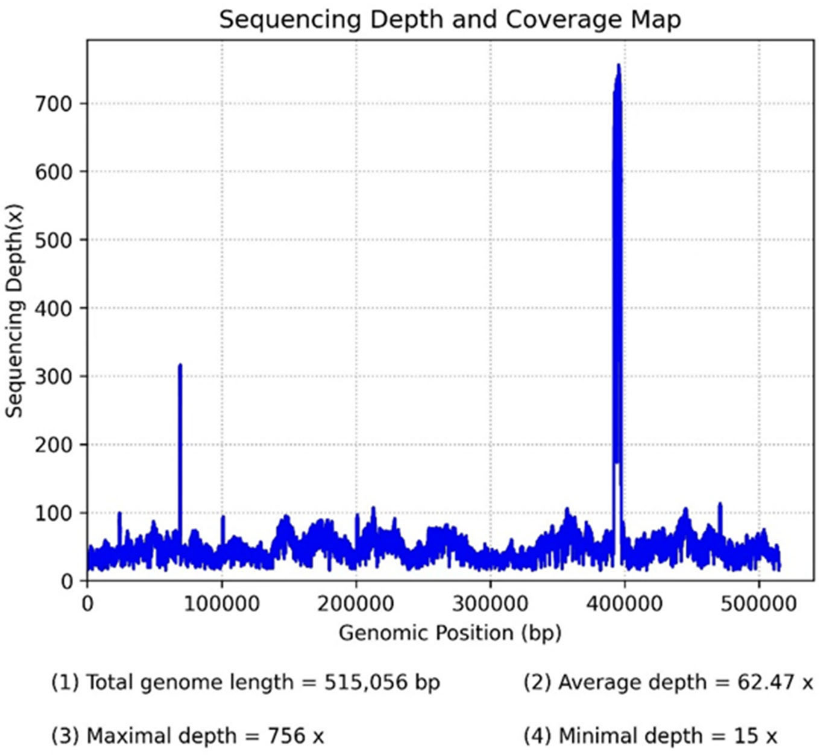

Figure S2. Quality and coverage of third-generation sequencing
